# Dose-dependent effects of human umbilical cord-derived mesenchymal stem cell treatment in hyperoxia-induced lung injury of neonatal rats

**DOI:** 10.3389/fped.2023.1111829

**Published:** 2023-03-08

**Authors:** Jing Xiong, Qing Ai, Lei Bao, Yuanshan Gan, Xiaoyu Dai, Mei Han, Yuan Shi

**Affiliations:** ^1^ Neonatal Diagnosis and Treatment Center of Children’s Hospital of Chongqing Medical University, Chongqing, China; ^2^ National Clinical Research Center for Child Health and Disorders, Chongqing, China; ^3^ Ministry of Education Key Laboratory of Child Development and Disorders, Chongqing, China; ^4^ China International Science and Technology Cooperation Base of Child Development and Critical Disorders, Chongqing, China; ^5^ Chongqing Key Laboratory of Pediatrics, Chongqing, China; ^6^ The Perfect Cell Biotechnology Co., Ltd, Chongqing, China

**Keywords:** hyperoxia, human umbilical cord-derived mesenchymal stem cells, bronchopulmonary dysplasis, neonate, inflammation

## Abstract

**Background:**

Mesenchymal stem cells (MSCs) are multipotent stromal cells that have been reported to possess great potential for the treatment of bronchopulmonary dysplasia (BPD).

**Objective:**

Our study aims to assess the effects of three different doses of intraperitoneal administration of human umbilical cord-derived MSCs (hUC-MSCs) on a hyperoxia-induced BPD model of newborn rat.

**Methods:**

Neonatal Sprague Dawley (SD) rats were reared in either hyperoxia (75% O2) or room air (RA) from postnatal days (PN) 1-14. At PN5, hUC-MSCs (1 × 106, 5× 106,or 1× 107 cells per pup) were given intraperitoneally to newborn rats exposed to 75% O2 from birth; the controls received an equal volume of normal saline (NS). At PN14, the lung tissues, serum, and bronchoalveolar fluid (BALF) were collected for histologic examination, wet/dry (W/D) weight ratio analysis, engraftment, myeoloperoxidase (MPO) activity analysis, cytokine analysis, and western blot analysis of protein expression.

**Results:**

Compared to rat pups reared in RA, rat pups reared in hyperoxia had a significant lower survival rate (53.3%) (*P* < 0.01). Hyperoxia-exposed rats exhibited pulmonary inflammation accompanied by alveolar-capillary leakage, neutrophile infiltration, augmented myeloperoxidase (MPO) activity, prominent alveolar simplification, and increased mean linear intercept (MLI), which was ameliorated by hUC-MSCs treatment. Increased oxidative stress and inflammatory cytokine production were also reduced. Importantly, the expression of Fas, an apoptosis-associated protein that was increasingly expressed in hyperoxia-exposed rats (*P* < 0.05), was downregulated after administration of hUC-MSCs (*P* < 0.05).

**Conclusions:**

Our results suggest that intraperitoneal administration of high number hUC-MSCs (1 × 107 cells) may represent an effective modality for the treatment of hyperoxia-induced BPD in neonatal rats.

## Introduction

Bronchopulmonary dysplasia (BPD) was first described by Northway in 1967 (1). It is a chronic lung disease in preterm infants resulting from supplemental oxygen or mechanical ventilation for respiratory distress syndrome (1). The incidence of BPD defined as the use of oxygen at 36 weeks' postmenstrual age or at discharge/transfer if before 36 weeks in neonates who survived to 36 weeks increases despite the tremendous advances in perinatal and neonatal medicine, including surfactant replacement therapy, antenatal steroids, and gentler ventilation techniques (2), and BPD remains the most common chronic disease in newborns with increased morbidity and mortality (3). The pathogenic mechanism of BPD is multifactorial and has also evolved, from striking fibrosis and cellular proliferation to arrested lung development, including inhibition of lung alveolar and vascular development (4, 5). Therapeutic strategies for the prevention and treatment of BPD including caffeine, vitamin A, surfactant, and steroids. In recent years, researches on mesenchymal stem cell (MSC)-based therapy have provided a novel approach for the prevention and treatment of BPD.

MSCs are multilineage cells with the ability to self-renew and differentiate into various cell types, which could be derived from bone marrow (BM), adipose, dental pulp, placenta, cord blood, and matrix (6, 7). They can modulate the immune response, activate cell proliferation, prevent apoptosis, promote angiogenesis, and improve regenerative responses and repair to protect tissues against a variety of injuries (8–11). These properties make MSCs an innovative potential cell-based therapy for regenerative medicine, especially for pediatric diseases (12). The most common source of MSCs in respiratory disease has been BM (13, 14). Nowadays, more attention has been drawn to umbilical cord-derived MSCs (UC-MSCs) due to their higher proliferation rate and that they can be extracted non-invasively. Besides, UC can generate MSCs with greater immunomodulatory potential than BM-MSCs.

In BPD animal models, MSCs can be delivered intravenously, intraperitoneally, intranasally, and intratracheally. In addition to the route of administration, the number of cells is also an important preclinical question. Various preclinical studies provided proof of concept for the lung-protective effect of MSCs. However, to date, the optimal number of cells and the route of MSC administration for the prevention and treatment of BPD are unknown. Therefore, this study aims to analyze the effect of three different numbers of human UC-MSCs (hUC-MSCs) *via* intraperitoneal injection on BPD model of rat pups and investigate the underlying mechanism.

## Materials and methods

### Characterization and analysis of hUC-MSCs

hUC-MSCs were obtained from Chongqing Perfect Cell Biotech Co., Ltd (Chongqing, China). The cells used in this study were followed by the International Society for Cellular Therapy Guidelines. The hUC-MSCs were maintained in Mesenchymal Stem Cell Basal Medium (MSCBM, Dakewe Biotech Corp., Shenzhen, China) supplemented with EliteGro^™^ (Biomedical Elitecell Corp., Woodway, TX, United States), at 37°C, saturating humidity and 5% CO_2_. They were characterized for the expression of specific cell surface markers (CD73, CD90, and CD105) through flow cytometry (Supplementary Figure S1A) and differentiation to osteogenic, adipogenic, and chondrogenic cells (Supplementary Figure S1B). The results revealed that hUC-MSCs used in this study were positive for typical MSC antigens (CD73, CD90, and CD105) but negative for hematopoietic antigens (CD34, CD45, and HLA-DR) (Supplementary Figure S1A). In addition, the hUC-MSCs showed the potential to differentiate into bone, fat, and cartilage (Supplementary Figure S1B).

### Animal model and experimental design

**Figure 1:**
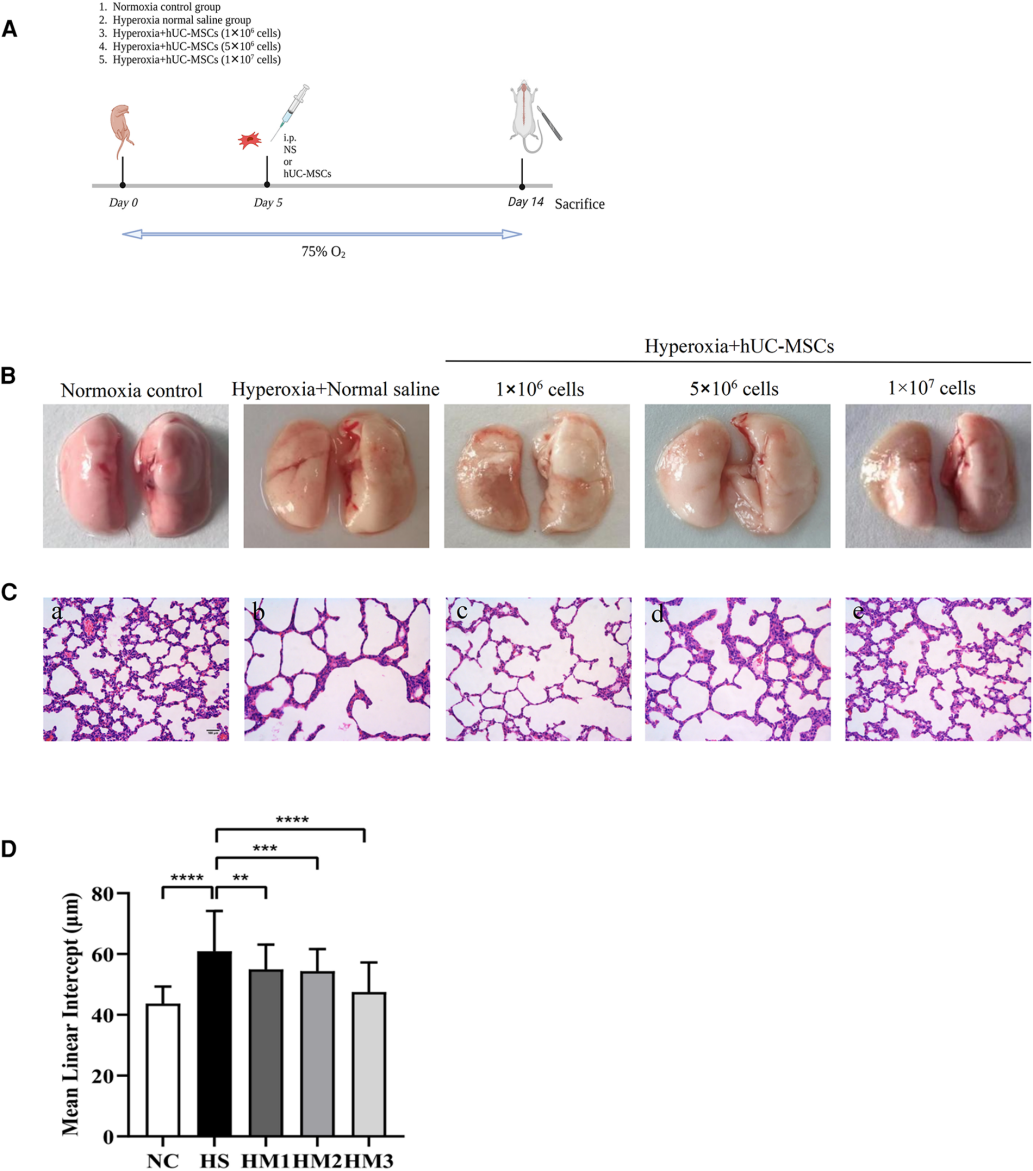
Human umbilical cord-derived mesenchymal stem cells (hUC-MSCs) restores hyperoxia-induced lung damages in bronchopulmonary dysplasia (BPD) of neonatal rats. (**A**) The schematic of hUC-MSCs procedure and study design; *n* = 14–16 per group. (**B**) Representative images of whole lung from all groups at postnatal day (PN) 14. (**C**) The histology of the lung in different groups identified using haematoxylin-eosin (HE) staining. Magnification, ×20 (scare bar = 100 µm). At PN 14, the HS group (b) demonstrated fewer and larger alveoli and heterogenous alveolar sizes as compared to the NC group (a). hUC-MSCs (1 × 106 cells, 5 × 106 cells, or 1 × 107 cells) treatment improved hyperoxia-induced damages in alveolar growth and morphological changes in HM1 (c), HM2 (d), and HM3 (e), respectively, in a dose-dependent manner. (**D**) Mean linear intercept (MLI) in 14-day-old rat pups. The rat pups reared in the HS group showed a remarkably higher MLI than did those of the NC group. hUC-MSCs (1 × 10^6^ cells, 5 × 10^6^ cells, or 1 × 10^7^ cells) remarkably reversed the hyperoxia-induced MLI increase. **P < 0.01, ***P < 0.001, and ****P < 0.0001. Data was presented as means ± SEM, *n* = 6–8 per group.

Sprague Dawley (SD) rats were purchased from the Experimental Animal Center of Chongqing Medical University and were raised in the Animal Laboratory Center of Pediatrics, in the Children's Hospital of Chongqing Medical University. All animal procedures and protocols were approved by the Ethics Committee of Chongqing Medical University. Time-dated pregnant SD rats were maintained in single cages at room temperature (between 20 and 24°C) with a 12/12 h light-dark cycle. Rats were provided with laboratory food and water *ad libitum* and allowed to deliver vaginally at term. Within 24 h of birth, the litters were pooled and distributed to the newly delivered mothers randomly. Newborn rat pups were randomly divided into five experimental groups: normoxia control group (NC), hyperoxia normal saline group (HS), hyperoxia with hUC-MSCs of 1 × 10^6^ cells group (HM1), hyperoxia with hUC-MSCs of 5 × 10^6^ cells group (HM2), and hyperoxia with hUC-MSCs of 1 × 10^7^ cells group (HM3). Rat pups of the NC group were maintained with a nursing mother rat in a single cage at room air (RA) throughout the experiment. Rat pups of the HS group were kept with a nursing mother in a sealed Plexiglas chamber in which the hyperoxia (oxygen concentration of 75%) was maintained until postnatal day (PN) 14. Humidity and environmental temperature were maintained at 50% and 24°C, respectively. Nursing dams were rotated between room air and the 75% hyperoxia groups every 24 h. Survival of rat pups in each group were observed daily during the experiment. All rat pups were sacrificed at PN14 under deep pentobarbital anesthesia (60 mg/kg, intraperitoneal) (Figure 1A). Lung tissues, bronchoalveolar lung fluid (BALF), and serum were collected for morphometric and biochemical analyses. Six to ten rat pups were used in each subgroup of analysis.

### Stem cell labeling

hUC-MSCs were transfected with lentivirus carrying GFP gene (lentiviral vector: pLVX-CMV-EGFP-PGK-PURO; virus titre: 2.6*10^9^ TU/ml) (Sangon Biotech CO., Ltd. Shanghai, China) at different MOI (10, 25, 50, 75) *in vitro* according to the manufacturer's protocol. The infection efficiency of hUC-MSCs was assessed at 48 h after transfected by lentivirus *via* flow cytometry.

### Assessment of hUC-MSCs engraftment

A suspension of 1 × 10^6^ cells of hUC-MSCs (the HM1 group) transfected by lentivirus carrying GFP gene in 200 µl normal saline was injected intraperitoneally per pup at PN5 after the pups had been already exposed to high oxygen. At 1, 12, 24, 48, and 72 h after injection, three pups from each timepoint were selected randomly and sacrificed after anesthesia. Left lung was fixed in 4% polyformaldehyde followed by embedding for paraffin sections and DAPI immunofluorescent staining. For immunofluorescence, after deparaffination and antigen retrieval process, sections were incubated with the primary antibody overnight at 4°C according to the manufacturer's instructions. Then the slides were washed and incubated with the appropriate secondary antibody at room temperature for 50 min in dark condition. After washing, tissue sections were incubated with DAPI solution, followed by spontaneous fluorescence quenching reagent. Primary antibody used in this study was anti-GFP (Abcam ab290). Microscopy detection and images were collected by Ortho-Fluorescent Microscopy.

### hUC-MSCs treatment

For hUC-MSCs transplantation, 1 × 10^6^ cells, 5 × 10^6^ cells, and 1 × 10^7^ cells in 0.2 ml normal saline were administered intraperitoneally at PN5 for HM1, HM2, and HM3 groups, respectively. For HS, an equal volume of normal saline was given intraperitoneally at PN5. After the procedure, the rat pups were allowed to recover from anesthesia and were returned to their nursing mothers. There was no mortality associated with the transplantation procedure.

### Lung histology and wet/dry (W/D) weight ratio

The whole left lung lobes of each animal were fixed in 4% paraformal dehyde, serially dehydrated in increasing concentrations of ethanol, and then embedded in paraffin. Two random sections from the lungs of each animal were stained with hematoxylin and eosin. Alveolarization was quantified using the mean linear intercept (MLI) based on the previous methods (15). Ten nonoverlaping fields of each section were acquired for morphological analysis by optical microscopy at a magnification of 20× (Nikon, Japan).

The lung W/D weight ratio was measured to assess the degree of pulmonary edema. The wet weight of the lung tissue was recorded before drying at 80°C for 48 h and reweighted until a stable dry weight was achieved. Then the lung W/D weight ratio was calculated.

### Analysis of bronchoalveolar lavage fluid (BALF)

After the animals were sacrificed at PN14, the lungs of pups were washed with phosphate-buffered saline (PBS) three times (five minutes each time) through the trachea cannula, and the washing fluid was collected and was centrifuged at 12,000 rpm for 15 min at 4°C. The supernatant was collected for determination of protein concentration using the BCA Protein Assay Kit (Beyotime, Shanghai, China). The total cell count was performed using the Countstar Automated Cell Counter (Ruiyu Biological Technology Co., LTD, Shanghai, China). Differential cell counts were made from centrifuged preparations stained with Wright-Giemsa staining, and at least 200 cells were counted in each pup.

### Analysis of myeloperoxidase (MPO) activity

Lung tissues were homogenized in normal saline with an appropriate proportion. Samples were then centrifuged at 12,000 rpm at 4°C for 20 min, and MPO activity in the supernatant was determined at 460 nm using a commercially available MPO activity colorimetric assay kit (Nanjing Jiancheng Bioengineering Institute, China).

### Analysis of MDA concentration

Lung tissues were lysed using lysis solution (MDA lysis buffer + BHT) (cat. no. ab118970; Abcam; Cambridge, UK). The cellular lysates were centrifuged at 13,000 rpm for 10 min at 4°Cand the supernatants were harvested. MDA concentration was determined using MDA kit according to the manufacturer's protocol (cat. no. ab118970; Abcam; Cambridge, UK).

### Assessment of HO-1 activity

HO-1 activity was estimated by using rat HO-1 ELISA kit (Ruixin Biological Technology Co., LTD, Quanzhou, China). It is a solid phase sandwich enzyme linked immunosorbent assay (ELISA), which uses a microtitre plate reader read at 450 nm. Activity of HO-1 was calculated from the plotted standard curve and expressed in U/L protein.

### Analysis of serum cytokine levels

A total of four serum cytokines, including (IL)-1β, IL-6, IL-10, and TNF-α, were detected simultaneously by using the MILLIPEX MAP Rat Cytokine/Chemokine Kit (EMD Millipore Corp., Billerica, MA, USA) according to manufacturer's protocol. Concisely, 200 µl assay buffer was added into each well of the plate for 10 min at room temperature with shaking and then removed. 25 µl of standard or control was added into the appropriate wells, followed by the add of assay buffer (25 µl) to the sample wells. Then the matrix solution (25 µl) was added to background, standards, and control wells with 25 µl of 1:2 diluted samples to sample wells. After mixing, 25 µl of beads were added to each well, and the plate was incubated for two hours at room temperature. After incubation, the well contents was removed, and the plate was washed 2 times. Following the addition of detection antibodies (25 µl) per well, the plate was incubated for one hour at room temperature with shaking. Then, 25 µl of Streptavidin-Phycoerythrin was added to each well, and plate was incubated for 30 min at room temperature with shaking. Well contents were gently removed and plat was washed two times. Then, 125 µl of Sheath Fluid or Drive Fluid was added into all wells, and the beads were resuspended for five minutes with shaking. Finally, the Median Fluorescent Intensity data were read on Luminex® and analyzed using a 5-parameter logistic or spline curve-fitting method for calculating cytokine concentrations in samples.

### Western blot analysis

Total protein of each sample was extracted from lung tissues by using radio immunoprecipitation assay (RIPA) analysis buffer and qualified by using a BCA protein assay kit (Beyotime, Shanghai, China). After denaturation, proteins (30 µg) were distinguished by 10% sodium dodecyl sulphate-polyacrylamide gel electrophoresis (SDS-PAGE) and transferred to a polyvinylidene difluoride (PVDF) membrane for blotting. After incubated with blocking buffer, the membranes were incubated with appropriate primary antibodies [anti-fas, Abcam, Cambridge, UK; horseradish peroxidase (HRP)-conjugatedβ-tubulin, ABclonal, Wuhai, China] overnight at 4°C. After washing, the membranes were incubated with HRP-conjugated secondary antibodies (Zen-bioscience, Chengdu, China), and the immunoreactive bands were visualised by enhanced chemiluminescence and analyzed with ImageJ software. The relative protein expressions were calculated after normalization with β-tubulin. Data were presented as expression level relative to the control group.

### Statistical analyses

Quantitative results were expressed as means ± SEM with *n* = 6 to 10 rats in each group, and statistical analysis was conducted by GraphPad Prism software version 8.0 (La Jolla, CA). Groups were compared with the two-tailed unpaired *t*-test and one- or two-way analysis of variance (ANOVA), as appropriate. *P*-values of <0.05 were considered statistically significant.

## Results

### Identification of infected hUC-MSCs

**Figure 2:**
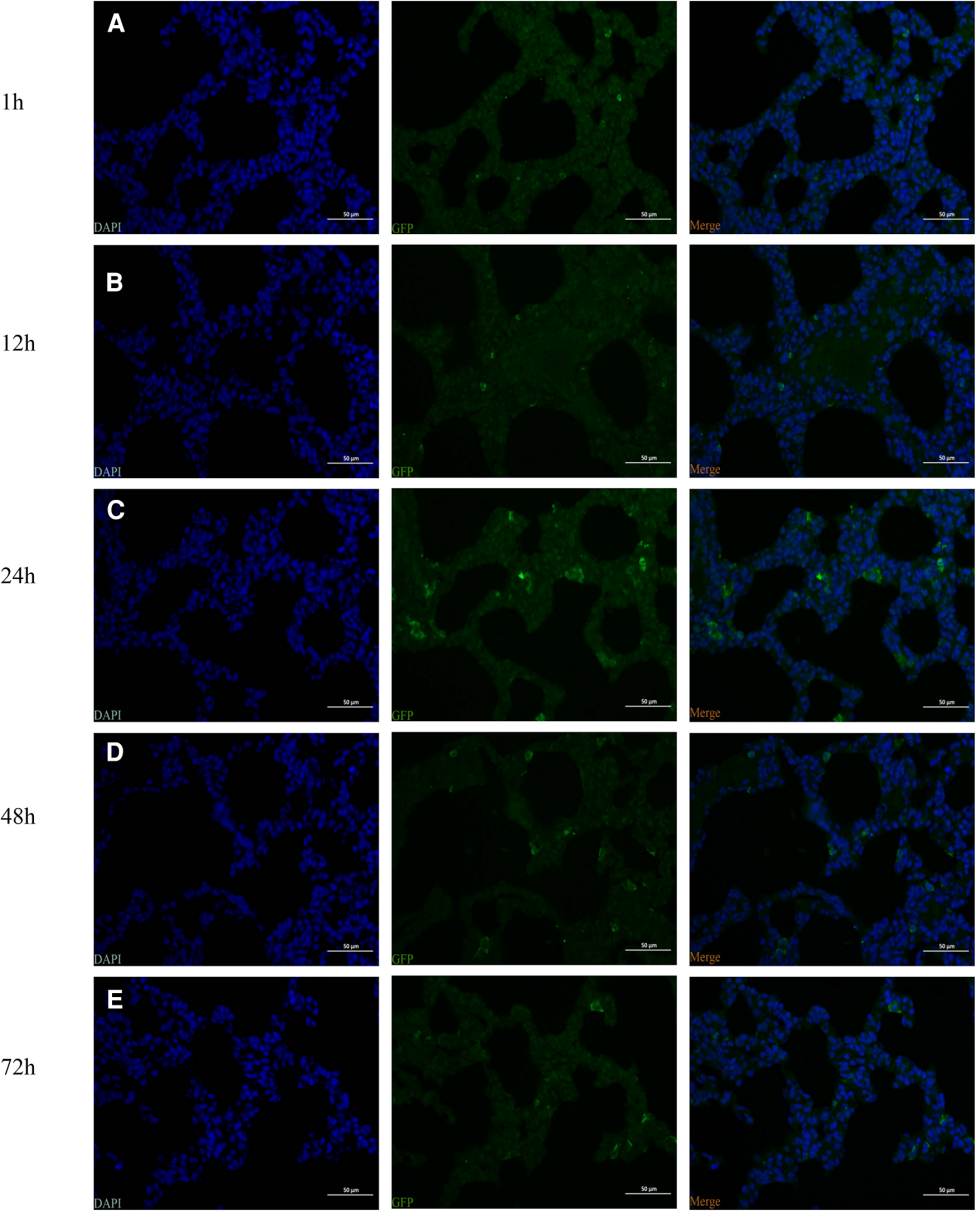
Identification of the intraperitoneal injection of human umbilical cord-derived mesenchymal stem cells (hUC-MSCs) (the HM1 group) by fluorescence microscopy. The green fluorescent protein (GFP)-labeled cells were identified by observing the distribution of GFP-positive cells. (**A**) 1 h, (**B**) 12 h (**C**) 24 h, (**D**) 48 h, and (**E**) 72 h. Magnification, ×400.

At 1, 12, 24, 48, and 72 h following injection of hUC-MSCs into rats, immunofluorescence for GFP in lung tissue was conducted. The images were captured with a camera system connected to a fluorescence microscope. GFP positive cells were found in the lungs of rat pups within 1 h after hUC-MSCs injection indicating that hUC-MSCs were first home to the lung after intraperitoneal infusion. One hour after injection, the signal increased and peaked at 24 h after injection, then decreased gradually. Even at 72 h after hUC-MSCs injection, GFP positive cells were still detected in the lung sections. These data revealed that the GFP-infected hUC-MSCs were able to migrate rapidly to lung tissue *in vivo* and function over time (Figure 2).

### hUC-MSCs administration increases survival rate in hyperoxia-induced BPD of neonatal rats

**Figure 3:**
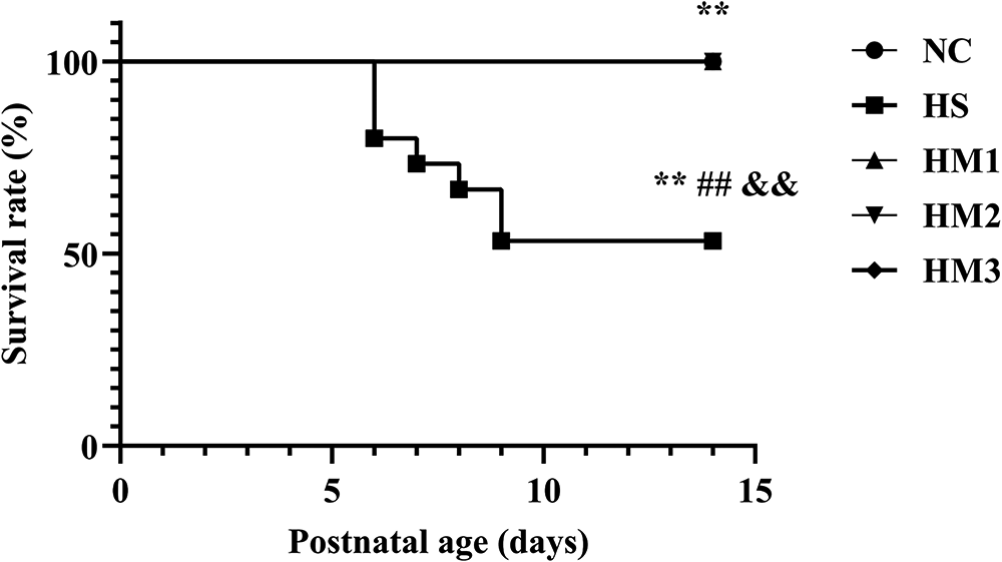
Human umbilical cord-derived mesenchymal stem cells (hUC-MSCs) improves survival rate of each group at postnatal day (PN) 14 showed in Kaplan-Meier survival curves. All the rat pups reared in the NC group (*n* = 14) and those reared in hyperoxia treated with human umbilical cord-derived mesenchymal stem cells (hUC-MSCs) (HM1, HM2, and HM3 groups) (*n* = 14–16) survived. The rat pups reared in the HS group (*n* = 14) had a significant reduced survival rate than did those reared in the NC group. hUC-MSCs improved the hyperoxia-induced decrease of survival rate, and the differences among the groups were statistically significant. **, ##, and &&*P* < 0.01.

Exposure to hyperoxia (HS) reduced the survival rate at 14 days of age (53.3%) compared to the 100% survival rate of NC at the same age. Administration with hUC-MSCs (HM1, HM2, and HM3) remarkably increased the survival rate in comparison with that in the BPD group (HS), with no death in the HM1, HM2, and HM3 groups (Figure 3).

### hUC-MSCs administration improves lung histology in hyperoxia-induced BPD of neonatal rats

Representative images of the whole lung from all groups at PN 14 are shown in Figure 1B. Representative lung sections stained with hematoxylin and eosin from newborn rats on PN14 are shown in Figure 1C. At PN14, compared to the NC group, the HS group demonstrated a histological pattern reminiscent of human BPD, characterized by severe impaired alveolar growth, as evidenced by fewer and larger alveoli and heterogenous alveolar sizes. And this was reflected in elevated MLI values compared with normoxia control rats (Figure 1D). After hUC-MSCs administration, hyperoxia-induced damage in alveolar growth and morphological changes were dramatically improved in HM1, HM2, and HM3, respectively, in a dose-dependent manner. A remarkably lower MLI was observed in the HM3 group (1 × 10^7^ cells per pup) as compared to that of the HM1 and HM2 groups.

### hUC-MSCs administration reduces pulmonary vascular permeability in hyperoxia-induced BPD of neonatal rats

**Figure 4:**
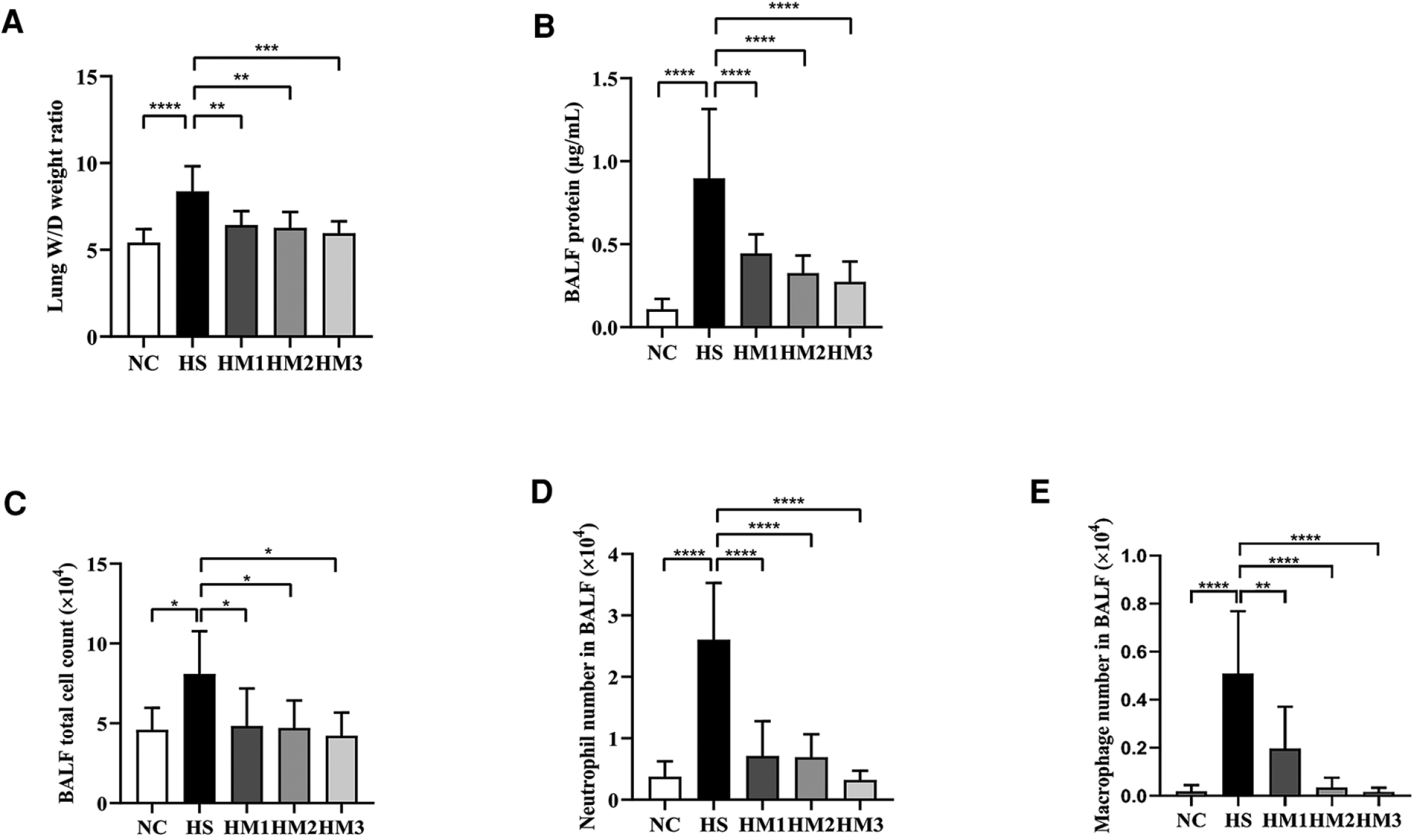
Human umbilical cord-derived mesenchymal stem cells (hUC-MSCs) reduces lung edema and lung inflammation in hyperoxia-induced bronchopulmonary dysplasia (BPD) of neonatal rats. (**A**) The lung wet/day (W/D) weight ratio, (**B**) Total protein concentration, (**C**) Total cell count, (**D**) Neutrophil number, and (**E**) Macrophage number in bronchoalveolar lavage fluid (BALF) of rat pups. The rat pups reared in the HS group showed a significantly increased lung W/D weight ratio, higher BALF total protein concentration, total cell count, and increased neutrophil and macrophage accumulation than did those reared in the NC group. hUC-MSCs (1 × 10^6^ cells, 5 × 10^6^ cells, or 1 × 10^7^ cells) reduced the hyperoxia-induced lung W/D weight ratio, total protein concentration, total cell count, neutrophil number, and macrophage number of BALF increases. *P < 0.05, **P < 0.01, and ****P < 0.0001. Data was presented as means ± SEM, *n* = 6–8 per group.

The BALF protein concentration and the lung W/D weight ratio are two commonly used indicators of pulmonary vascular permeability. A significant increase in the BALF protein concentration (Figure 4A) and the lung W/D weight ratio (Figure 4B) was observed in the hyperoxia-induced BPD rats when compared with those of the NC group, and this level was decreased by hUC-MSCs treatment in HM1, HM2, and HM3 group, in a dose-dependent manner. These results suggest that hUC-MSC treatment attenuates lung edema in hyperoxia-induced BPD rats.

### Impact of hUC-MSCs on differential cell counts of BALF in hyperoxia-induced BPD of neonatal rats

The total cell number in BALF was counted using the Countstar Automated Cell Counter, and differential cell counts were evaluated using centrifuged preparations stained with Wright-Giemsa staining (Figures 4C–E). The numbers of total cells, neutrophils, and macrophages in BALF were significantly increased in the HS group compared to the NC group, and those in the hUC-MSCs-treated group (HM1, HM2, and HM3) were significantly lower than those in the HS group, in a dose-dependent manner.

### hUC-MSCs administration reduces neutrophil infiltration into the lungs in hyperoxia-induced BPD of neonatal rats

**Figure 5:**
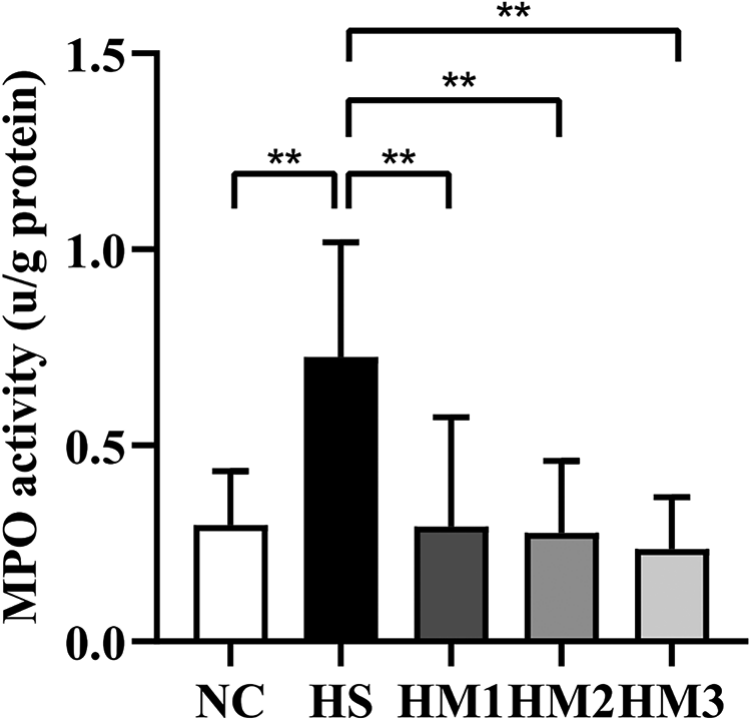
Human umbilical cord-derived mesenchymal stem cells (hUC-MSCs) reduces myeloperoxidase (MPO) activity of the lung tissue. The rat pups reared in the HS group yielded significantly higher MPO activity than did those reared in the NC group. hUC-MSCs (1 × 10^6^ cells, 5 × 10^6^ cells, or 1 × 10^7^ cells) reduced the hyperoxia-induced MPO activity increase. **P < 0.01. Data was presented as means ± SEM, *n* = 6–8 per group.

To measure the degree of neutrophil infiltration in the lung, MPO activity was detected. Compared with that in the NC group, lung MPO activity in the HS group was dramatically increased (Figure 5). The increased MPO activity observed in the HS group was significantly attenuated in HM1, HM2, and HM3 groups, and this attenuation was most significant in HM3 group, next in HM2 and HM1 groups.

### hUC-MSCs administration alleviates oxidative stress in lung tissues of hyperoxia-induced BPD of neonatal rats

**Figure 6:**
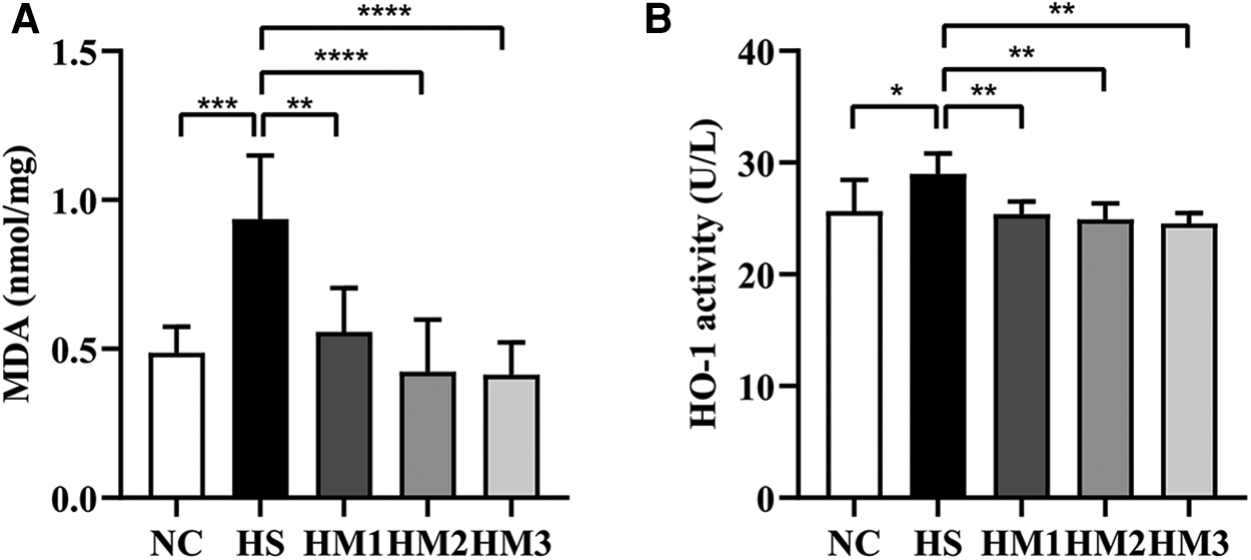
Human umbilical cord-derived mesenchymal stem cells (hUC-MSCs) reduces the level of MDA concentration (**A**) and HO-1 activity (**B**). The rat pups reared in the HS group showed significantly higher level of MDA concentration and HO-1 activity than did those reared in the NC group. hUC-MSCs (1 × 10^6^ cells, 5 × 10^6^ cells, or 1 × 10^7^ cells) diminished the hyperoxia-induced MDA level and HO-1 activity increases. *P < 0.05, **P < 0.01, ***P < 0.001, and ****P < 0.0001. Data was presented as means ± SEM, *n* = 6-8 per group.

MDA is a marker of free radical activity. Extensive evidence has shown that oxidative stress markedly increases MDA level. Similarly, heme oxygenase (HO)-1 is an essential enzyme in heme catabolism physiologically that possesses anti-inflammatory properties and suppresses oxidative stress. In the present study, the HS group revealed a substantially higher levels of MDA concentration and HO-1 activity in lung tissues compared to the NC group. Elevated MDA level and HO-1 activity induced by hyperoxia exposure were significantly diminished upon hUC-MSCs administration in hyperoxia-exposed neonatal rats, in a dose-dependent manner (Figures 6A,B).

### hUC-MSCs administration regulates cytokine levels in hyperoxia-induced BPD of neonatal rats

**Figure 7:**
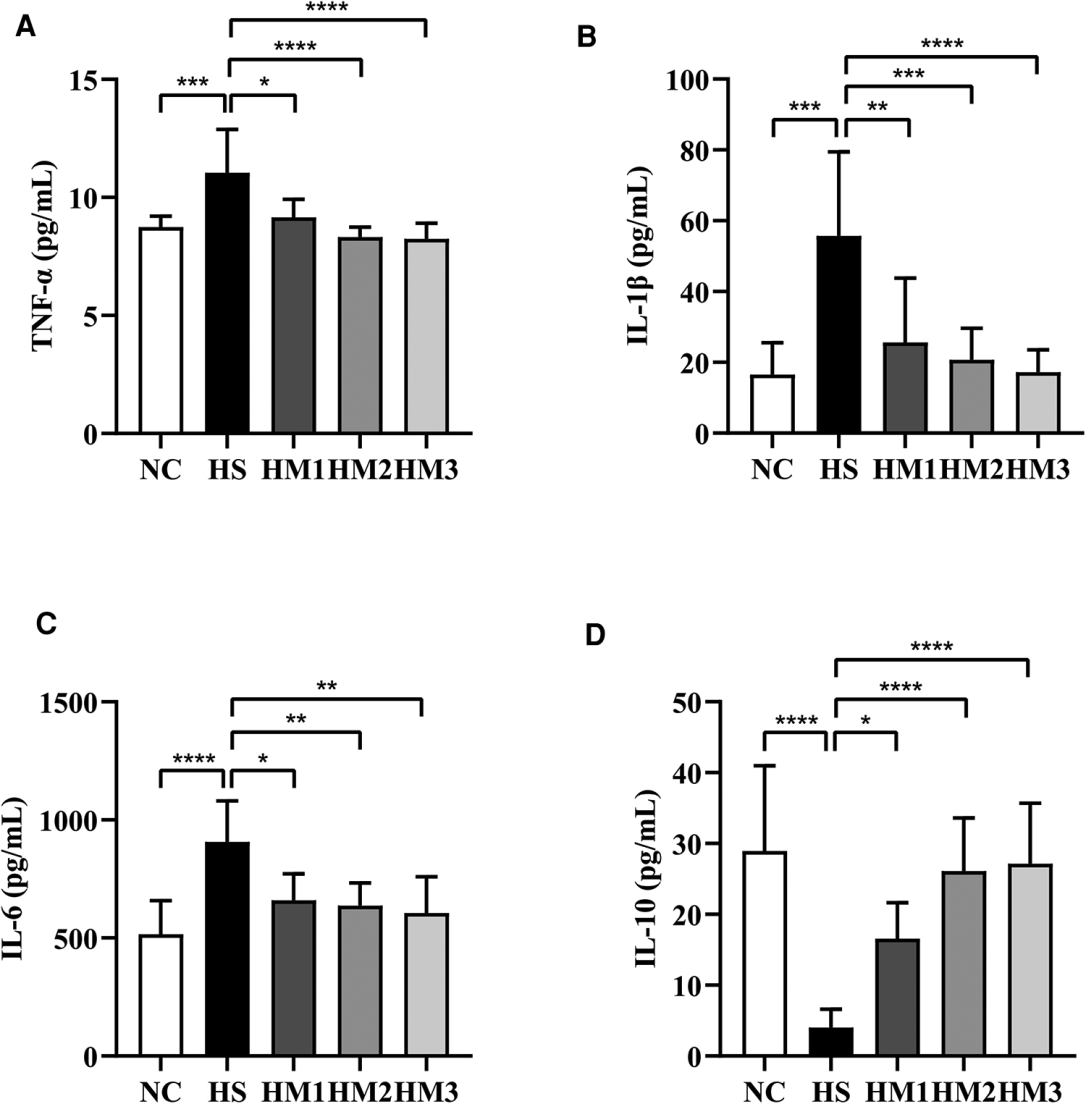
Human umbilical cord-derived mesenchymal stem cells (hUC-MSCs) reduces proinflammatory cytokine levels and increases anti-inflammatory cytokine level in rat pups. The rat pups reared in the HS group showed significantly higher levels of IL-1β, IL-6, and TNF-α and lower level of IL-10 than did those reared in the NC group. hUC-MSCs (1 × 10^6^ cells, 5 × 10^6^ cells, or 1 × 10^7^ cells) reduced the hyperoxia-induced IL-1β, IL-6, and TNF-α level increases and augmented the hyperoxia-induced IL-10 level decrease. *P < 0.05, **P < 0.01, ***P < 0.001, and ****P < 0.0001. Data was presented as means ± SEM, *n* = 6–8 per group.

As depicted in Figures 7A–D, we found that the expression of the pro-inflammatory cytokines TNF-α, IL-6, and IL-1β in serum of the BPD rats were remarkably increased compared to those of the NC group. This hyperoxia-induced increases in TNFα, IL-1β, and IL-6 levels were significantly attenuated in HM1, HM2, and HM3 group in a dose-dependent manner. Conversely, we observed a decrease in anti-inflammatory cytokine IL-10 expression in rats exposed in hyperoxia, while the decreased IL-10 expression observed in the HS group was significantly augmented in HM1, HM2, and HM3 group, in a dose-dependent manner.

### hUC-MSCs administration downregulates the apoptosis-associated protein in hyperoxia-induced BPD of neonatal rats

**Figure 8:**
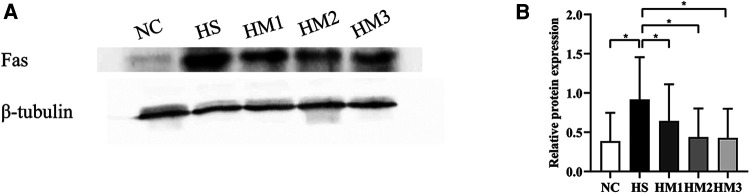
Human umbilical cord-derived mesenchymal stem cells (hUC-MSCs) inhibits Fas protein expression in rat pups. (**A**) The protein expression of Fas in the lung tissue evaluated by western blot. (**B**) Statistical analysis of protein expression of Fas. The rat pups of the HS group showed significantly higher protein expression of Fas than did those of the NC group. hUC-MSCs (1 × 10^6^ cells, 5 × 10^6^ cells, or 1 × 10^7^ cells) downregulated the hyperoxia-induced Fas protein level increase. *P < 0.05. Data was presented as means ± SEM, *n* = 5 or 6 per group.

The expression level of apoptosis-associated protein Fas in lung tissue was determined by Western blotting (Figure 8). There was a significant increased level of Fas in the HS group compared to the NC group, and the increased expression level was downregulated by hUC-MSCs treatment in a dose-dependent manner. These results suggested that hUC-MSCs may suppress the apoptosis of hyperoxia-induced BPD in rats by modulating the expression level of Fas.

## Discussion

BPD develops due to impaired alveolarization in preterm infants and extends into childhood with severe respiratory problems. Various animal models have been developed and continue to be refined with the aim of recapitulating the pathological pulmonary hallmarks noted in the lungs of neonates with BPD. Plenty of preclinical studies commonly use rats to make BPD models. Reasons are various, including relatively short gestation times allowing quick studies on lung development and that the term rat lungs present a similar development stage with human preterm neonates between 24 and 28 gestation weeks, which allows them a proper model for developmental lung injury (16).

MSCs are hypoimmunogenic and more tolerated by hose immune system than other types of stem cells (17), which makes them a great cell source for transplantation in the treatment of BPD. Numerous preclinical studies have shown that treatment with MSCs can alleviate neonatal lung injury in animal models mimicking BPD (18). In particular, the discrepancy concerning the partial effect of MSCs could be explained by numerous differences, including the cell concentration at harvesting, species, hyperoxia level, and the administration route. And studies have reported that administration of MSCs *via* intraperitoneal injection may be as effective as *via* intratracheal injection or intravenous injection (12, 18). Given that MSCs have been transplanted intrapetitoneally with a good safety record and effectiveness, we chose to administer MSCs *via* the intraperitoneal route in our study. Due to technical limitations, a maximum of 1 × 10^7^ cells were transplanted. The results revealed that intraperitoneal delivery of hUC-MSCs improved the survival rate, restored the airway structure caused by exposure to hyperoxia, exhibited decreased alveolarization as evidenced by increased MLI, reduced lung edema, reduced cellular infiltration and total protein in BALF, alleviated oxidative stress, modulated levels of inflammatory cytokines, and downregulated level of apoptosis-related protein Fas in a dose-dependent manner, and that 1 × 10^7^ donor cells seems to be optimal to maximize protective effects in the experimental model and setting.

MSCs labeled by transfection with a GFP-carrying lentivirus were administered intraperitoneally to rats at PN5 to enable visualization of localization. GFP-positive cells were seen in the lung tissue of all rats that received labeled cells one hour after transplantation, mainly in the airway, alveolar epithelial cells, and alveolar septum. Even 72 h after transplantation, GFP-positive cells were still seen in the lung tissues of all rats. This explains the potential mechanism by which treatment with hUC-MSCs intraperitoneally improves lung development through engraftment.

The oxidant/antioxidant balance is a vital component in the pathogenesis of ALI (19). MDA is the main product of lipid peroxidation and is identified as a biological marker of oxygen stress injury (20, 21). Substantial evidence demonstrated that heme HO-1 is an inducible enzyme with potent anti-oxidant, anti-inflammatory, and anti-apoptotic properties (22, 23). Multiple preclinical studies have shown that HO-1 regulates the protective response in hyperoxia-induced injury (24–26). In the present study, we found that hUC-MSCs administration remarkably downregulated MDA and HO-1 expression in lung tissue, which suggests that hUC-MSCs alleviate BPD *via* modulating the oxidative/antioxidative balance.

A plenty of proinflammatory factors could be activated during oxidative stress, including IL-1β, IL-6, and TNF-α (27–29). Overproduction of these factors promotes chronic inflammation, which leads the development of BPD. Preclinical studies have reported that the inhibition of inflammatory factors has beneficial effects on lung injury by decreasing lung inflammation and oxidative stress in neonatal rats. Oncel et al. reported that the inhibition of TNF-α decreased the MDA levels, which helps the lung development and pulmonary vascularization (30). Besides, studies also showed that IL-1β and IL-6 could be used as biomarkers for monitoring ALI (28, 29, 31). As one of the most important anti-inflammatory cytokines, IL-10 is known to inhibit the synthesis of proinflammatory cytokines. Study indicated that the progression of ALI is associated with decreased expression and secretion of IL-10 (32). In this study, we observed that hUC-MSCs significantly decreased the levels of these proinflammatory cytokines TNF-α, IL-1β, IL-6, and MPO and increased the expression of anti-inflammatory cytokine of IL-10 in the serum of BPD rats, which is consistent with the previous studies (33, 34), and probably suggests that inflammatory responses mediated by neutrophils, oxidative stress, and proinflammatory cytokines play an important role in the pathogenesis of BPD (35, 36). Many previous studies have reported that the protective effects of MSCs transplantation against hyperoxia-induced lung injury are mainly mediated by paracrine potency rather than regenerative mechanisms (12, 37, 38). Various paracrine mediators are known to be protective against hyperoxia-induced lung injury, including increased inflammation, oxidative stress, apoptosis, and impaired angiogenesis and alveolarization (38, 39). In addition, a growing corpus of studies have highlighted that MSCs can modulate T-cell-mediated immunological responses (40). In the present study, the protective effects of hUC-MSCs treatment against hyperoxia-induced lung injury were positively correlated with reduced levels of proinflammatory cytokines of TNF-α, IL-1β, and IL-6, total protein and cells accumulation including neutrophils, reduced MPO activity, MDA concentration, and HO-1 activity, in a dose-dependent manner. Our results suggest that intraperitoneal delivery of at least 1 × 10^6^ cells of hUC-MSCs is necessary to induce paracrine effects in hyperoxia-induced lung injury of newborn rats.

The Fas-mediated cell death pathway represents typical apoptotic signaling in many cell types (41–43), and Fas signaling also was involved in hyperoxia-induced apoptosis (44). A number of investigations demonstrated that acute lung injury increases the expression of Fas in epithelial cells (45–47). Besides, activation of the Fas signaling triggers inflammatory responses in the lung, including cytokine release from epithelial cells *via* activation of protein kinases (45). Prevention of Fas expression results in the reduction of apopsis (48). Some studies have demonstrated that hyperoxia has started apoptosis in animal models *via* Fas and death receptor-mediated apoptotic pathway (49, 50). And MSCs therapy possesses protective effects on apoptosis mediated through the death receptor pathway (51). In our study, the protein expression of Fas was markedly up-regulated in the HS group, which was remarkably down-regulated by hUC-MSCs in a dose-dependent manner. Our results suggest that hUC-MSCs transplantation saves lungs from pulmonary injury by suppressing the upregulation of hyperoxia-triggered apoptosis, along with the reduced inflammation.

## Conclusion

In conclusion, our data indicated that intraperitoneal administration of hUC-MSCs significantly ameliorated the hyperoxia-induced lung injury, including reversing decreased alveolarization and increased inflammation. The intraperitoneal delivery of 1 × 10^7^ cells was optimal to achieve effective anti-inflammatory, anti-oxidative, and anti-apoptotic effects. However, even if this dose might be appropriate for newborn rats, it might not be suitable for humans on a kilogram basis. Further studies are needed to explore the optimal dose of hUC-MSCs for the treatment of BPD infants.

## Data Availability

The original contributions presented in the study are included in the article/Supplementary Material, further inquiries can be directed to the corresponding author/s.
