# Supplementary figures and images for: Dose-dependent effects of human umbilical cord-derived mesenchymal stem cell treatment in hyperoxia-induced lung injury of neonatal rats
Source: Front Pediatr. 2023 Mar 8;11:1111829. doi: 10.3389/fped.2023.1111829 (PMC10032376; doi:10.3389/fped.2023.1111829)

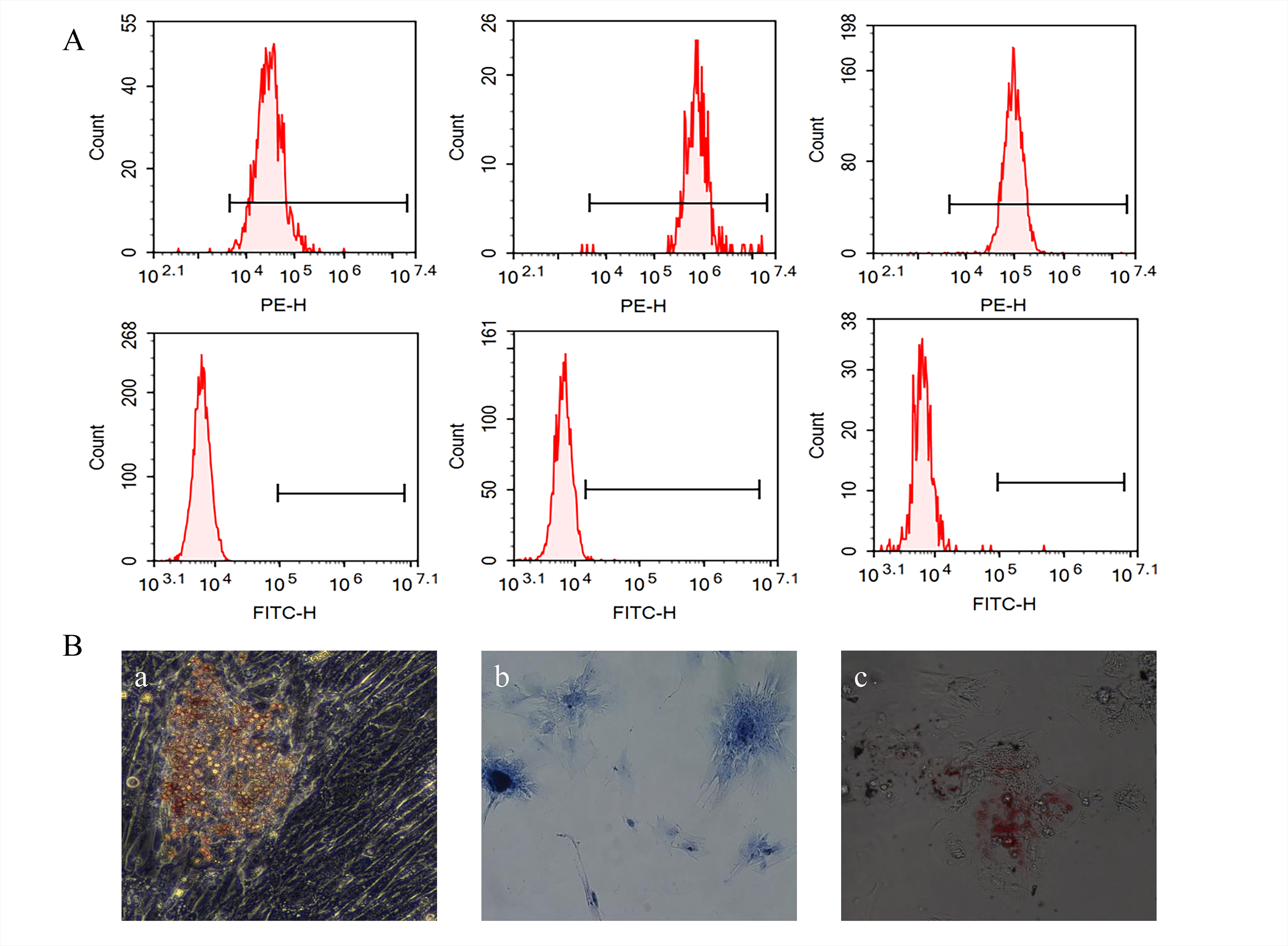

Supplement: Supplementary file 1 [file Image1.tif]
